# A Case of Acute Dilatation of the Stomach

**Published:** 1909-07-10

**Authors:** 


### Surgery.

### A CASE OF ACUTE DILATATION OF THE STOMACH.

Acute dilatation of the stomach is an occurrence of great rarity, especially if it appears to be idiopathic; that is to say if the symptoms come on suddenly in an individual who was up to that time in his normal state of health. In the great majority of these cases the dilatation of the stomach is secondary to some other serious disorder. It has been known, for instance, to follow upon operations on the pelvic viscera in females and upon the gall-bladder in both sexes; and cases have been recorded in which it appeared as a complication of ulcerative endocarditis and a subphrenic abscess respectively.

The following notes are those of a case of primary acute dilatation of the stomach which was under the care of the writer. The patient, a French girl aged 25, could only give a vague history of the onset of her trouble. She said that three days before seeking advice she had been seized with diarrhœa, and that next day she had a sudden acute pain all down the left side of the stomach, which had persisted. Since the onset of this pain she had vomited everything she had taken. She had occasionally vomited after her meals in the past, but had never had hæmatemesis nor had she been prone to indigestion. She was a vegetarian.

The only possible indiscretion of diet to which the patient could in any way attribute her symptoms was the taking of some pork gravy just before the attack began. No other member of the household who partook of this dish suffered any inconvenience. When she was first seen she looked ill and was in severe abdominal pain referred to the left half of the abdomen. Her aspect was that of a case of peritonitis, with sunken eyes and an anxious countenance. Her pulse 160 and feeble, and her temperature 96° F.

The upper three-fourths of the abdomen was distended and appeared to be occupied by an enormous oblong mass whose long axis was vertical. There was no movement on respiration in the distended portion of the abdominal wall, and there was distinct resistance on palpation. On percussion, not only was the mass itself resonant, but the liver dulness was completely absent, except for a small area in the right axillary line. A vaginal examination was made, but revealed no abnormality of the pelvic viscera.

An immediate operation was decided upon, a provisional diagnosis of perforated gastric ulcer being made, as everything seemed to point to this rather than to any less usual condition.

The abdomen was accordingly opened through a vertical incision by splitting the fibres of the left rectus abdominis muscle in its upper half. Immediately on opening the peritoneum a portion of a large rounded swelling with distended veins upon its surface came into view. This was resonant on percussion.

The incision was enlarged to admit the hand, and the shape and relations of the swelling carefully palpated. It was then discovered that the tumour was an enormously distended stomach which was bent upon itself in a U-shaped manner, the most dependent portion resting upon the fundus uteri. The upper pole of the swelling was pushing the liver well up out of reach under the costal arch. A small opening was made into the organ: at first a rush of gas made its escape, and this was followed by a quantity of greenish-yellow fluid, which drained away for a considerable time. The amount of this fluid evacuated was measured and found to exceed six-and-a-half pints.

Under the influence of the anæsthetic the patient showed immediate improvement as soon as the distension of the stomach was relieved. The wound in the stomach was sewn up with Lambert sutures, and the organ was then systematically palpated. No gross abnormality could be discovered; that is to say there was no sign of a recent or old-standing ulcer at the pyloric end and certainly no evidence of pyloric stenosis. The abdominal wound was sewn up in layers, an indiarubber tube being inserted at the lower end as a precautionary measure.

After the operation the patient was allowed nothing to take for some days except tea. She still had pain for 24 hours after the operation, but no vomiting at all. The tube was removed on the third day. There was no evidence that the stomach was dilating again, *i.e.* its percussion note was normal in distribution. On the sixth day the patient was allowed to have milk, and pounded fish on the ninth day. On the tenth day the stitches were removed. The wound healed by first intention. The rest of her convalescence was uneventful.

There were several points of interest about the case. In the first place, should one have been able to arrive at a correct diagnosis? In the writer's opinion the only feature which was unlike a perforated gastric ulcer was that the abdominal distension was sharply limited at its lower level, as if the abdominal wall were pressed out by a localised tumour rather than by the distended intestine in a late stage of peritonitis.

Apart from these considerations the history in this case was most suggestive of a perforated gastric ulcer—the abdominal facies, the resistance of the upper abdomen, and the almost complete absence of liver dulness associated with a quick, feeble pulse, presented a syndrome almost pathognomonic of advanced peritonitis.

Of course, if the diagnosis can be made absolutely certain, it is obviously incorrect treatment to open the abdomen, since acute dilatation of the stomach can be cured by the passage of the stomach tube and keeping the patient in a prone position. But if there be the slightest doubt, the writer's opinion is that the abdomen should be opened without delay. After all, it is a far graver error to allow a case of peritonitis to die with the abdomen unopened than to do an unnecessary laparotomy in such a case as the one under discussion.

